# Alkaloidal Extracts from *Avicennia africana* P. Beauv. (Avicenniaceae) Leaf: An Antiplasmodial, Antioxidant, and Erythrocyte Viable

**DOI:** 10.1155/2024/4541581

**Published:** 2024-01-09

**Authors:** Mustapha A. Ahmed, Elvis O. Ameyaw, Francis A. Armah, Patrick M. Fynn, Isaac Asiamah, George Ghartey-Kwansah, Felix K. Zoiku, Ebenezer Ofori-Attah, Christian K. Adokoh

**Affiliations:** ^1^Department of Biomedical Sciences, School of Allied Health Sciences, University of Cape Coast, Cape Coast, Ghana; ^2^Small Animal Teaching Hospital, SVM, CBAS, University of Ghana, Legon, Accra, Ghana; ^3^Department of Pharmacotherapeutics and Pharmacy Practice, School of Pharmacy and Pharmaceutical Sciences, University of Cape Coast, Cape Coast, Ghana; ^4^Department of Chemistry, School of Physical Sciences, University of Cape Coast, Cape Coast, Ghana; ^5^Department of Epidemiology, Noguchi Memorial Institute for Medical Research, College of Health Sciences, University of Ghana, Legon, Accra, Ghana; ^6^Department of Clinical Pathology, Noguchi Memorial Institute for Medical Research, College of Health Sciences, University of Ghana, Legon, Accra, Ghana; ^7^Department of Forensic Sciences, School of Biological Science, University of Cape Coast, Cape Coast, Ghana

## Abstract

**Background:**

The emergence of drug-resistant parasites impedes disease management and eradication efforts. Hence, a reinvigorated attempt to search for potent lead compounds in the mangroves is imperative.

**Aim:**

This study evaluates *in vitro* antiplasmodial activity, antioxidant properties, and cytotoxicity of *A. africana* leaf alkaloidal extracts.

**Methods:**

The *A. africana* leaves were macerated with 70% ethanol to obtain a total crude extract. Dichloromethane and chloroform-isopropanol (3 : 1, v/v) were used to extract the crude alkaloids and quaternary alkaloids from the total crude. The antiplasmodial activities of the alkaloidal extracts were performed against 3D7 *P. falciparum* chloroquine-sensitive clone via the SYBR Green I fluorescence assay with artesunate serving as the reference drug. The alkaloidal extracts were further evaluated for antioxidant properties via the total antioxidant capacity (TAC), the total glutathione concentration (GSH), the DPPH (2,2-diphenyl-1-picrylhydrazyl) assay, and the ferric-reducing antioxidant power (FRAP) methods. The cytotoxic activity of the alkaloidal extracts was tested on erythrocytes using a 3-(4,5-dimethylthiazol-2-yl)-5-diphenyltetrazolium bromide-MTT assay with little modification. The phytocompounds in the alkaloidal extracts were identified via gas chromatography-mass spectrometry (GC-MS) techniques.

**Results:**

The total crude extract showed good antiplasmodial activity (IC_50_ = 11.890 *µ*g/mL). The crude and quaternary alkaloidal extracts demonstrated promising antiplasmodial effects with IC_50_ values of 6.217 and 6.285 *µ*g/mL, respectively. The total crude and alkaloidal extracts showed good antioxidant properties with negligible cytotoxicity on erythrocytes with good selectivity indices. The GC-MS spectral analysis of crude alkaloidal extracts gave indole and isoquinoline alkaloids and several other compounds. Dexrazoxane was found to be the main compound predicted, with an 86% peak area in the quaternary alkaloidal extract.

**Conclusion:**

The crude and quaternary alkaloidal extracts exhibited antiplasmodial activities and ability to inhibit oxidative stress with negligible toxicity on erythrocytes. This may be good characteristics to avoid oxidative stress related to *Plasmodium* infection in the treatment of malaria.

## 1. Introduction

Malaria remains a fatal infection with public health significance. The infection is caused by plasmodial species such as *Plasmodium vivax*, *P. malariae*, *P. ovale*, *P. knowlesi*, and *P. falciparum*. *Plasmodium falciparum* is predicted to cause 99.7% of all the malaria cases in Africa [1]. The infection is spread by the bites of infected female *Anopheles* mosquitoes [2]. The World Health Organization (WHO) projected 241 million malaria cases in 2020, with 627,000 mortalities, the vast majority of which were children under the age of five [2]. Africa continues to be the infection hotspot, accounting for 94% of the global disease and mortality burden [2].

In several countries, including Ghana, artemisinin-based combination drugs are still the first-line treatment regime for uncomplicated malaria [3]. However, concerns about emerging and widespread antimalarial drug resistance pose a serious drawback for malaria control. *Plasmodium falciparum* has been reported to resist quinoline-based drugs such as chloroquine [4] and artemisinin [5, 6]. Resistance to these and other antimalarial drugs, as well as the lack of an effective vaccine [7], necessitates a renewed effort to find new, effective, and affordable antimalarial agents from a variety of natural resources available.

Natural compounds found in plants have been suggested to possess infinite therapeutic potential [8], with many of these agents having promising antimalarial properties. Both quinines, isolated from Cinchona bark [9], and artemisinin, developed from the *Artemisia annua* plant, are two well-known antimalarial lead compounds [10]. But quinine is often used to treat uncomplicated malaria in pregnancy, severe malaria, treatment failure in artemisinin-based combination treatment (ACT) therapies, and malaria in children under five [11, 12]. Consequently, various alkaloids have been reported to show varying biological activities, including notable antimalarial effects. Several classes of these alkaloids with antimalarial activity have been identified [13]. Bekeo and collaborators reported that antimalarial drugs with an alkaloidal base could be a good alternative to ACTs in Ghana [9].

Several mangrove plants used in folk medicine have been proposed to have tremendous therapeutic potential [14]. Preliminary research revealed that their extracts exhibit a diverse array of biological activities, including antifungal, antibacterial, anticancer, antidiabetic, and antiviral properties, due to the presence of bioactive metabolites [15]. *Avicennia africana*, which is associated with the West African mangrove, has been shown to have a high concentration of alkaloids and saponins [16, 17] and a variety of pharmacological properties, including antimalarial effects, in both *in vitro* and *in vivo* assays [18].

In malarial pathogenesis, haemoglobin degradation by malarial parasites generates redox-active by-products such as free haem [19], hydrogen peroxide, and hydroxyl radicals in *P. falciparum*-infected RBCs [20], which cause oxidative insult to host cells. This could suggest an association between parasite pathophysiology and free radical generation, as well as a drop in antioxidant levels in the host system [21]. The oxidative stress caused by malaria infections may cause significant pathological damage to important organs in humans, such as the liver and spleen, as well as cognitive impairment. It has been revealed that the use of antimalarial drugs frequently leaves residues of this damage following therapy, as evidenced by memory impairment after cerebral malaria [22]. Hence, plants or compounds with antimalarial effects and antioxidant properties could help in malaria management and possibly prevent infection aftereffects. In view of this, alkaloidal extracts from *A. africana* leaves found in mangroves may have the potential to revolutionise the battle against malaria.

This study investigated the antiplasmodial activity of the 70% ethanol total crude extract and alkaloidal extracts of *A. africana* leaf against the chloroquine-sensitive clones of 3D7 parasites using the SYBR Green I fluorescent assay. In addition, the antioxidant activities of the extracts as well as their alkaloids were determined by utilising various quantitative techniques. The tetrazolium-based colorimetric (MTT) assay technique was used to test the cytotoxic effects of the alkaloids and total crude extract on human erythrocytes. Furthermore, the phytocompounds of the alkaloidal isolates were identified using GC-MS analysis.

## 2. Materials and Methods

### 2.1. Collection and Authentication of the Plant

**Figure 1:**
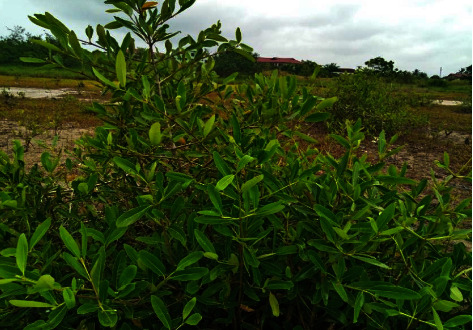
Photograph of *Avicennia africana* whole plant and leaves. Source: fieldwork, Ahmed et al. [17, 18].

The leaves of the *A. africana* plant, also locally called “Dwira Akyinim,” in Akan were sampled from a mangrove forest area called “Iture,” a coastal community near Elmina, Cape Coast, in the Central Region of Ghana (Figure 1). The plant sample was identified and validated by a botanist at the Department of Environmental Studies' herbarium at the School of Biological Sciences, University of Cape Coast. A voucher number (CC3096) was assigned to the plant specimen for future reference.

### 2.2. Plant Extraction

The *A. africana* leaves were cleaned with tap water, shade-dried, and ground into a fine powder. The dried and powdered leaves (6.5 kg) were extracted by cold maceration in 70% ethanol (3 x 1.5 L) for 72 h [23]. The combined extracts were concentrated under reduced pressure in a Rotary thin-film evaporator (R-114 SABITA) to afford a greenish-gummy crude extract (AAE, 412.18 g, 6.34% w/w). All solvents and reagents used in the total crude and alkaloidal extractions were of analytical-grade quality and obtained from Merck Chemical Supplies (Darmstadt, Germany) and Sigma-Aldrich (Germany).

### 2.3. Extraction of Alkaloids from the Total Crude Extract (AAE) of *A. africana*

The crude extract (412.18 g) was dissolved in 30% acetic acid and filtered. The clear acidic solution was extracted with chloroform (3x500 mL). The chloroform layer was discarded and the aqueous layer was basified to a pH of 10.5 using 25% aqueous ammonia and extracted with dichloromethane (3x150 mL). The dichloromethane layer was dried using anhydrous magnesium sulphate and evaporated under reduced pressure to dryness to obtain a light brownish crude (AAA, 5.23 g, 1.26% w/w,) [24]. The screening of this extract using Dragendoff reagent, Mayer's reagent, and 3% Ce (NH_4_)_2_SO_4_ in 85% H_3_PO_4_ revealed the presence of alkaloids. Once again, the aqueous layer was extracted with chloroform- isopropanol mixture (3 :1 v/v, 3x250 mL). The chloroform-isopropanol layer was concentrated to give a light brownish solid (AAQ, 6.13 g, 1.49%) [24, 25]. This light brownish solid gave a positive test with the Mayer's reagent [25, 26].

### 2.4. Parasite Cultivation

The efficacy of the alkaloidal extracts (AAA and AAQ) and total crude (AAE) was tested against a 3D7 *P. falciparum* clone (chloroquine sensitive) obtained from the Immunology Department, Noguchi Memorial Institute for Medical Research, University of Ghana. The asexual forms of *P. falciparum* were preserved in continuous cultures by employing techniques suggested by Rapoport and Holden [26], with minor modifications. The parasites were cultured in 2% packed cell volume (O^Rh^ ^+^ noninfected human erythrocytes) and maintained in a complete culture medium (CPM). The medium is composed of RPMI-1640 supplemented with 5.94 *µ*g/L HEPES, 5 *µ*g/L AlbuMAX II, 50 mg/L hypoxanthines, and 2.1 *µ*g/L sodium carbonate (NaHCO_3_). All chemicals and reagents utilized in this study were procured from Sigma-Aldrich (Germany) and QualiChem's Lab Reagents (India). The incubation conditions were 3% O_2,_ 4% CO_2,_ 93% N_2_, and 37°C. The culture media were changed daily to ensure that parasitaemia was greater than 5%. A solution of 5% sorbitol was used to treat cultures and incubated for 48 hours to attain synchrony of ring-stage parasites. The parasites were subcultured to obtain 1% parasitaemia before being used in assays.

### 2.5. Antiplasmodial Activity of Crude and Alkaloidal Extracts

The stock solutions (1000 *μ*g/mL) of the alkaloidal extracts (AAA and AAQ) and total crude extract (AAE) were filter-sterilized using a 0.2 *μ*m Millipore filter. A working solution (100 *μ*g/mL) was achieved by diluting the stock solution 10-fold. It was further diluted to attain concentrations ranging from 100 to 0.39 *μ*g/mL. An aliquot of 100 *µ*L of each of the nine dilutions was plated in duplicate in each well of a 96-well coastal plate. A standard antimalarial reference drug, artesunate (15 ng/ml working concentration), was serially diluted up to the 9^th^ concentration (15−0.06 ng/mL) and plated alongside. Each well received 100 *μ*L of parasite culture (1% parasitaemia at 2% haematocrit). All the other extracts were taken through the same procedure. The plates were gassed for 5 minutes in a modular incubation chamber with 90% N_2_, 5% CO_2_, and 5% O_2_ before being kept at 37°C for 72 h for incubation.

After 72 h of incubation, the cultures were treated with 100 *µ*L of lysing buffer (SYBR Green I fluorescent), which is composed of 0.08% Triton-X 100, 5 mM EDTA, 20 mM Tris-Cl (pH 7.5), and 0.008% saponin as suggested by Johnson et al. [27], with slight modifications. The lysing buffer was treated carefully to avoid creating bubbles in the wells. Before reading the plates, they were left at room temperature for 30–60 minutes in the dark. The plates were read at 470 and 520 nm using the FLUOstar OPTIMA Fluorometer plate reader with Control Software version 2.20.

### 2.6. Screening of Alkaloidal and Crude Extracts for Cytotoxic Effect

The cytotoxic properties of the alkaloidal extracts and total crude extracts were tested on erythrocytes using a slightly modified version of the 3-(4,5-dimethylthiazol-2-yl)-5-diphenyltetrazolium bromide-MTT assay as described by Ayisi et al. [28]. A total of 100 *µ*L of extracts (twofold serial dilution) ranging in concentration from 6.25 to 100 *µ*g/mL were dispensed (in duplicate) into separate wells of a 96-well microtiter plate. A volume of 100 *µ*L of noninfected erythrocytes was added to each well and incubated for 3 days at 37°C in a humidified incubator (5% CO_2_ and O_2_). Following that, each well received 20 *µ*L of 7.5 mg/mL MTT in PBS and was kept for 2 h. After that, an aliquot (150 *µ*L) of culture media was removed from each well and discarded. The plates were treated with 200 *µ*L of Triton X-100 in acidified isopropanol to dissolve any formazan that had formed. The plates were maintained in the dark at room temperature for 24 hours before being read at 570 nm with a plate reader. The concentrations at which the extracts kill 50% of the cells (CC_50_ values) were determined using Microsoft Excel 2016 software to create a graph of the extract concentrations versus percentage mean cell viability with dose-response curves. The CC_50_ values were compared to standard values to find out if the total crude and the alkaloidal extracts were harmful to cells. In addition, to find the selectivity indices (SI), the ratios of toxic concentrations of extracts (CC_50_) to effective bioactive doses (IC_50_) are used to determine the amount of extract that inhibits or kills the parasites with no toxicity.

### 2.7. Antioxidant Assays

#### 2.7.1. Evaluation of the Total Antioxidant Capacity (TAC) of Alkaloidal and Crude Extracts

The total antioxidant capacity of alkaloidal extracts (AAA and AAQ) and total crude extract (AAE) was determined using the phosphomolybdenum assay with minor modifications [29]. In this method, 50 *µ*L of AAA, AAQ, and AAE were mixed with 500 *µ*L of reagent solution (4 mM ammonium molybdate, 28 mM sodium phosphate, and 0.6 M sulfuric acid). The compositions were kept for one hour at 95°C before being cooled and read at 695 nm with a FLUOstar Optima (BMG Labtech) against a blank (50 *µ*L of DMSO). Ascorbic acid (a standard antioxidant) in DMSO with varying concentrations (1, 0.5, 0.250, 0.125, 0.0625, 0.0312, and 0.0156 *μ*g/mL) was used to create the calibration curve. The total antioxidant activity was expressed as mg/g of ascorbic acid.

#### 2.7.2. Scavenging Activity of Alkaloidal and Crude Extracts on DPPH Radical

The antioxidant activities of alkaloidal extracts were assessed using a slightly modified DPPH (2,2-diphenyl-1-picrylhydrazyl) assay [30]. Ascorbic acid (0.5 mg/mL in methanol) was diluted 2-fold, which served as the positive control. The total crude (AAE), crude alkaloids (AAA), and quaternary alkaloids (AAQ) (2.5 mg/mL in methanol) were individually constituted to generate seven distinct concentrations. The reaction began with the transfer of 100 *µ*L of each total crude or alkaloidal extract or ascorbic acid into a 96-well plate, followed by the addition of 100 *µ*L of a 0.5 mM DPPH solution into the wells. The absorbances at 517 nm were measured with a plate reader (Tecan Infinite M200 Pro, Austria) after the mixture was kept for 20 minutes. Methanol was used as a negative control. The experiments were conducted in triplicates. The antioxidant activities of the extracts were expressed as a percentage of free-radical scavenging activity (%FRSA), which was calculated as follows:

(1) $$\begin{array}{c} \left[\frac{\left(\text{Ao}-\text{Ae}\right)}{\text{Ao}}\right]\times 100, \end{array}$$

where Ao = absorbance of the blank solution and Ae = absorbance of the test (extract) solution or the standard (ascorbic acid).

The effective concentration at 50% free radical scavenging activity (EC_50_) was found by plotting a graph of the percentage of free radical scavenging activity vs. the concentration of the sample.

#### 2.7.3. Assessment of Glutathione GSH Concentration

The approach proposed by Cereser et al. [31], with minor modifications, was used in the assessment of the glutathione (GSH) concentration inherent in the AAE, AAA, and AAQ. The reaction solutions were made up of 10 *µ*L of the crude extract and alkaloidal extracts (5 mg/mL in DMSO). In addition, 180 *µ*L of GSH buffer (100 mM NaH_2_PO_4_, 1N NaOH, 5 mM EDTA, and pH 8.0) and 10 *µ*L of O-phthaldehyde (0.75 mM) were used for the reaction. The mixture, together with the GSH standard solution (0.0001563–0.1 mg/mL in DMSO (2-fold serial dilution)), was kept at room temperature for 15 minutes. The fluorescence was read at 350 nm (the excitation wavelength) and 420 nm (the emission wavelength). The tests were run in triplicate. A calibration curve for the GSH standard solution, with a regression equation (*y* = 651473*x* + 103.69, *R*^2^ = 0.998) was made to figure out how much glutathione was present in the extracts. The total glutathione in the extracts was expressed as glutathione equivalent (GSH).

#### 2.7.4. Ferric-Reducing Antioxidant Power (FRAP) Assay

The FRAP test, as proposed by Benzie and Strain [32], was used in this study with a minor modification using a 96-well microplate. In a 10 : 1:1 ratio, 2.5 mL of 20 mmol/L FeCl_3_ solutions, 2.5 mL of 10 mmol/L TPTZ solution, and 25 mL of 300.0 mmol/L acetate buffer were mixed to make the FRAP reagent. The AAE, AAA, and AAQ (20 *μ*L) were mixed violently together with 180 *μ*L of the FRAP reagent. In the presence of antioxidants, the complex compound ferric tripyridyltriazine (Fe^3+^-TPTZ) is reduced to its ferrous tripyridyltriazine (Fe^2+^-TPTZ) form, resulting in an intense blue colour that can absorb maximally at a wavelength of 593 nm.

### 2.8. Profiling of *A. africana* Extracts Using Gas Chromatography-Mass Spectrometry (GC-MS)

The profiles of alkaloidal extracts (AAA and AAQ) from *A. africana* leaf (70% ethanol extract, AAE) were analysed on an Agilent 7890 B GC with an Agilent Technologies GC sampler 80 (Agilent Technologies, CA, USA). The device was equipped with an MS Agilent 7000°C triple quadrupole with a column size of 30 m + 10 m EZ Guard × 0.25 mm internal diameter-fused silica capillary coated with VF-5 ms (0.25 mm film) from Agilent or equivalent. The temperatures of the injector (in splitless mode) and the MSD transfer line were set to 280°C and 325°C, respectively. The extract was dissolved in methanol and injected at an initial column temperature of 70°C for 25 min. The system temperature was increased up to 150°C (3°C/min), 200°C (8°C/min), and 280°C (2.133 minutes). Helium was the carrier gas with a constant flow rate of 2.25 mL/minute, with nitrogen serving as the collision gas with a constant flow rate of 1.5 mL/minute. The septum purge was performed at a rate of 30 mL/minute for 0.75 minutes at a pressure of 27.5 psi. The mass detector could scan at m/z values ranging from 50 to 550. To identify the phytocomponents in the extracts, an injection volume of 2 *µ*L (10 mg/mL in acetonitrile) of the samples was used for analysis. The compounds detected were identified by correlating the various peaks produced with the mass spectral library NIST 2014 (National Institute of Standards and Technology, Mass Spectral Library) [33]. The confirmation and characterisation of the alkaloidal metabolites were done using isotopic fit ratios (iFit) and mass accuracy. This fragmentation pattern analysis provides important information regarding the number of isotopes in the molecule to facilitate molecular formula determination which ensured the dependability and correctness of the phytocompounds identified.

### 2.9. Analysis of Data

The tests were conducted in triplicate. The data were shown as the mean ± standard deviation (SD). The IC_50_ values were obtained from graphs of dose-response curves through the application of GraphPad Prism 5.0 version software (GraphPad Software Inc., San Diego, CA). The CC_50_ and EC_50_ values were also derived from a dose-response curve using Microsoft Excel 2016. The student *t*-test was employed for analysis, and statistical significance was set at *p* < 0.05.

## 3. Results

### 3.1. Total Crude and Alkaloidal Extracts Yield

**Figure 2:**
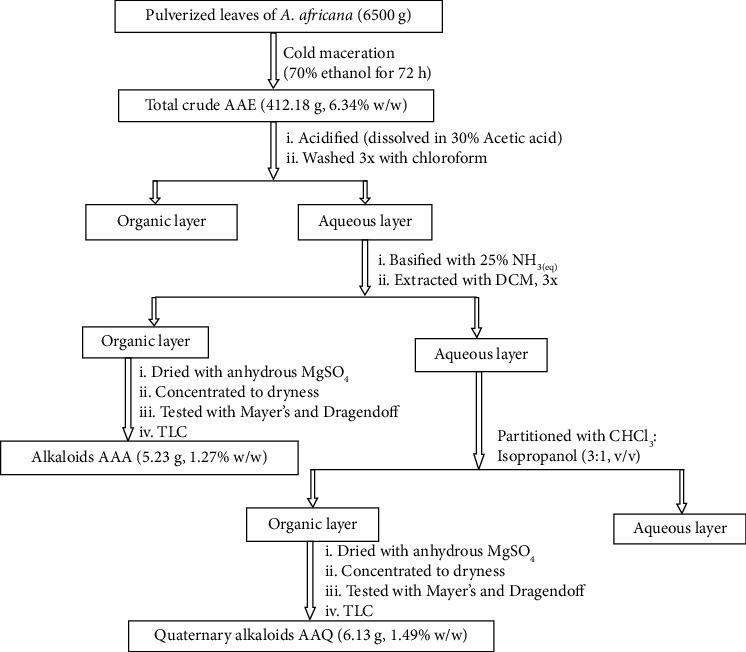
Schematic diagram for the extraction of alkaloids from *A. africana*.

The 70% v/v ethanol cold maceration of 6.5 kg of pulverised leaf material yielded 412.18 g (6.34% w/w) of the total crude extract (AAE). To allow the extraction of the alkaloidal components, the crude extract was treated with aqueous acid and washed with chloroform. The aqueous layer was made basic to convert the alkaloids back into their neutral forms and subsequently extracted with DCM to afford the alkaloidal extract (AAA: 5.23 g, 1.26% w/w). The aqueous layer was further partitioned into chloroform-isopropanol (3 : 1 v/v) to give a light brownish solid believed to be quaternary alkaloids (AAQ: 6.13 g, 1.49%), as shown in Figure 2 [25, 26]. The basified aqueous extract with DCM was preliminary confirmed by positive Mayer's and Dragendoff tests. After chloroform-isopropanol extraction, the basified aqueous extract was tested for quaternary alkaloids and found to be positive for Mayer's test [25, 26].

### 3.2. Antiplasmodial Effects of *A. africana* Total Crude and Alkaloidal Extracts

The antiplasmodial effects of the total crude and crude alkaloidal extracts from the leaves of *A. africana* were tested against 3D7 *P. falciparum* strains, and the results are shown in Table 1. The IC_50_ values for the extracts (AAE, AAA, and AAQ) were 11.890 *µ*g/mL, 6.217 *µ*g/mL, and 6.285 *µ*g/mL, respectively. The IC_50_ value for the control drug, artesunate, was 0.9 × 10^−3^ *µ*g/mL. Previous research suggests that extracts with IC_50_s below 5 *µ*g/mL have “very active” antiplasmodial action, while those between 5 and 50 are “active,” 50 and 100 are “weakly active,” and those above 100 are “inactive.” [34]. Similarly, Kamaraj et al. [35] also suggested that plant extracts with IC_50_s of less than 10 *µ*g/mL are classified as having “promising” antiplasmodial activity. They also said that IC_50_s between 10 and 20 *µ*g/mL, 20 and 40 *µ*g/mL, 40 and 70 *µ*g/mL, and more than 70 *µ*g/mL had “moderate,” “good,” “marginally potent,” and “poor” antiplasmodial activity, respectively. The latter antiplasmodial activity score categorization was used in this study. Based on the IC_50_ values obtained for AAE, AAA, and AAQ, the extracts demonstrated moderate to promising activity against 3D7 *P. falciparum* parasite clones.

### 3.3. Cytotoxicity of Alkaloidal Extracts of *A. africana*

**Figure 3:**
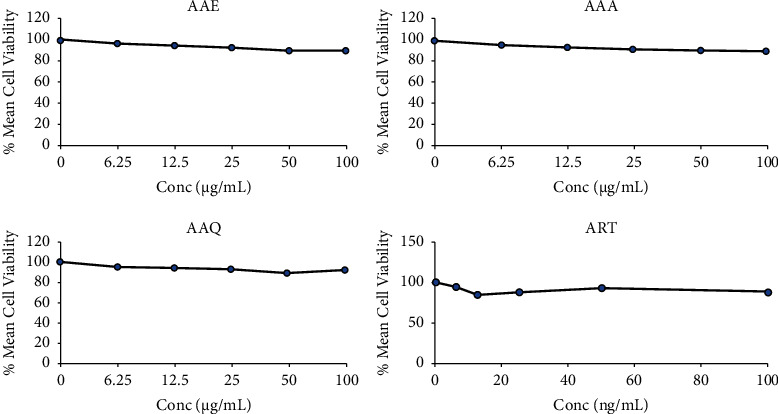
Erythrocytes' survival following the subjection of *A. africana* total crude (AAE), crude alkaloids (AAA), quaternary alkaloids (AAQ), and artesunate (ART) to uninfected red blood cells (RBCs). The experiments were triplicated (*n* = 3), with cytotoxic effect (CC_50_) values greater than 100 for all the extracts as well as the control drug (ART).

The outcome of the erythrocytes' survival is shown in Figure 3 after RBCs were subjected to different concentrations of the alkaloidal extracts. The cell survival rate of the alkaloidal extracts was similar to that of the artesunate reference drug. Also, both the crude and alkaloidal extracts showed good selectivity for 3D7 parasites, as indicated by their selectivity indices of >2 (Table 1).

### 3.4. Antioxidant Activity of Total Crude and Alkaloidal Extracts

**Figure 4:**
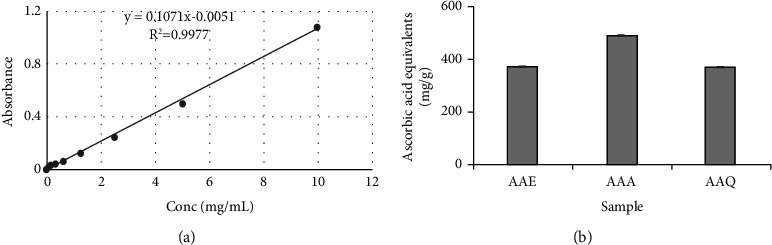
(a) TAC standard calibration curve and (b) total antioxidant capacity of *A. africana* total crude and alkaloidal extracts AAE, AAA, and AAQ. Data are presented as the mean value ± standard deviation SD (*n* = 3).

The alkaloidal extracts and the total crude of *A. africana* yielded an appreciable amount of overall antioxidant activity. The AAE and AAQ had total antioxidant capacities (TAC) of 375.506 ± 0.047 and 373.638 ± 0.040 mg/g, respectively (Figure 4). The AAA had the highest TAC value, at 494.39 ± 0.058 mg/g ascorbic acid equivalent. In this test, both the total crude and the alkaloidal extracts contained adequate quantities of antioxidants required to neutralise free radicals at varying concentrations.

**Figure 5:**
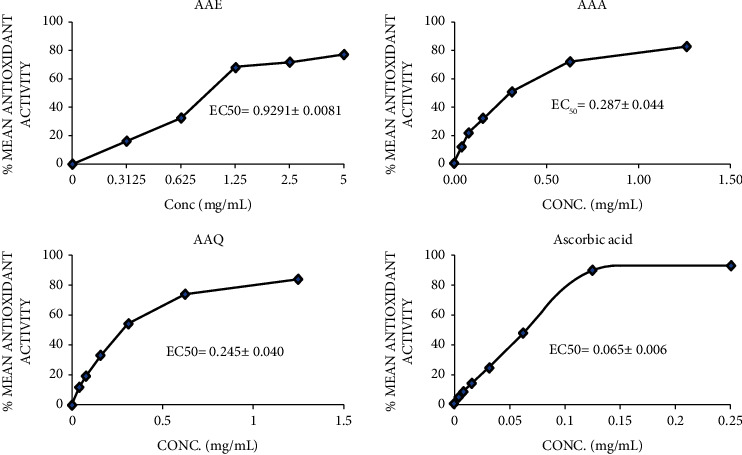
The percentage mean antioxidants activities (DPPH free-radical scavenging) of *A. africana* total crude (AAE), crude alkaloids (AAA), quaternary alkaloids (AAQ), and ascorbic acid. Data show the mean ± standard deviation SD for three repeated runs (*n* = 3) (*p* < 0.0001).

The scavenging abilities of AAE, AAA, and AAQ crude extracts compared to that of ascorbic acid (control) are presented in Figure 5. The various effective concentrations (EC_50_) of the extracts were found to be 0.929 ± 0.008 mg/mL, 0.287 ± 0.044 mg/mL, 0.245 ± 0.040 mg/mL, and 0.065 ± 0.006 mg/mL, respectively. Compared to ascorbic acid (0.065 ± 0.006), the alkaloidal extracts (AAA and AAQ) had the strongest scavenging activities (*p* < 0.0001), with the total crude (AAE) extract having the least. This suggests that the alkaloidal components of the plant are mainly responsible for its radical scavenging activity.

**Figure 6:**
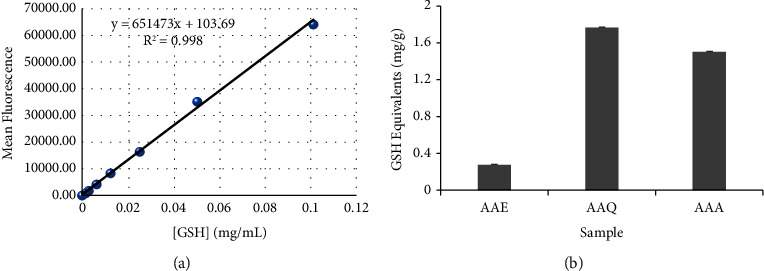
(a) GSH standard curve and (b) reduced GSH concentrations in AAE, AAQ, and AAA of the *A. africana* plant. The data are shown as the mean ± standard deviation (SD) (*n* = 3). The experiments were triplicated.

**Figure 7:**
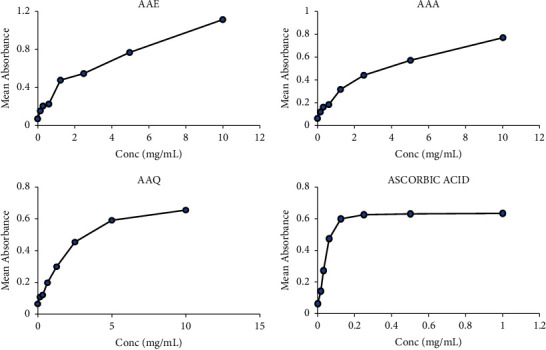
Ferric-reducing antioxidant power (FRAP) of the total crude AAE (1.722 ± 0.268 mg/mL) and alkaloidal extracts AAA (3.568 ± 0.759 mg/mL), AAQ (3.386 ± 0.015 mg/mL), and ascorbic acid (0.077 ± 0.005 mg/mL).

The total glutathione (GSH) content inherent in AAE, AAA, and AAQ extracts yielded varying concentrations. The total GSH levels in the total crude and the alkaloidal extracts were AAE: 0.269 ± 0.0001, AAQ: 1.764 ± 0.0001, and AAA: 1.495 ± 0.0002 mg/g GSH equivalent, as shown in Figure 6. The AAQ and AAA extracts were found to have higher levels of glutathione concentrations (*p* < 0.0001) than the AAE. Similarly, the ferric-reducing antioxidant power (FRAP) of AAE, AAA, and AAQ extracts showed a considerable variance in EC_50_s (AAE, 1.722 ± 0.268 mg/mL; AAA, 3.568 ± 0.759 mg/mL, and AAQ, 3.386 ± 0.015 mg/mL) compared to ascorbic acid (0.077 ± 0.005 mg/mL) (Figure 7). The EC_50_s of the antioxidant activities of the extracts were concentration dependent. AAE exhibited the highest power-reducing activity in comparison to ascorbic acid, followed by AAQ and AAA.

### 3.5. GC-MS Analysis of Compounds from the Alkaloidal Extracts of *A. africana*

**Figure 8:**
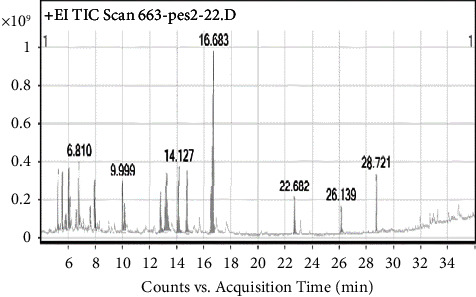
GC profile of the crude alkaloidal extracts (AAA) of *A. africana* shows the retention time (min) of the compounds on the *X*-axis, and the *Y*-axis represents the percentage (%) of peak area.

**Figure 9:**
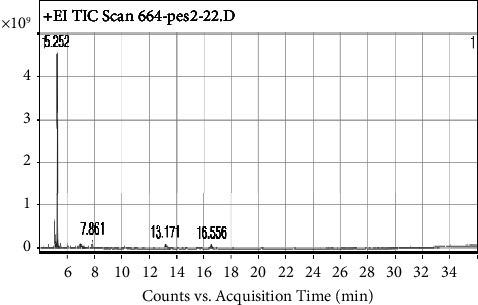
GC-MS profile of the quaternary alkaloidal extract (AAQ) of *A. africana* shows the retention time (min) of the compounds on the *X*-axis, and the *Y*-axis represents the percentage (%) of peak area.

GC-MS analysis of the extract showed that AAA had 19 peaks and AAQ had 7 peaks, showing the presence of different phytocompounds. As presented in Figure 8 and Table 2, the peak with a retention time of 16.683 minutes was assigned to gramine. Gramine is an aminoalkylindole alkaloid (C_11_H_14_N_2_; MW-174.24 gmol^−1^) (M^+^) and had the highest peak area (31.97%) of all the compounds found in the AAA extract. On the other hand, dexrazoxane (C_11_H_16_N_4_O_4_; MW-268.27 gmol^−1^) with a retention time of 5.252 minutes was the major compound with the highest percentage composition (90.7%) in the AAQ extract, with traces of other six compounds as shown in Figure 9 and Table 3.

#### 3.5.1. Identification and Characterization of Alkaloidal Metabolites

The respective MS spectrum of each alkaloidal metabolite was generated, and on the basis of isotopic fit ratios (iFit) close to zero and, more importantly, that the overall MS accuracy was within 5 mDa, the molecular formulae were computed [36]. The Dictionary of Natural Products online database (https://dnp.chemnetbase.com) was used to identify compounds. The molecular formulae of these respective alkaloids were carefully chosen on the 5 mDa mass accuracy range scale.

**Figure 10:**
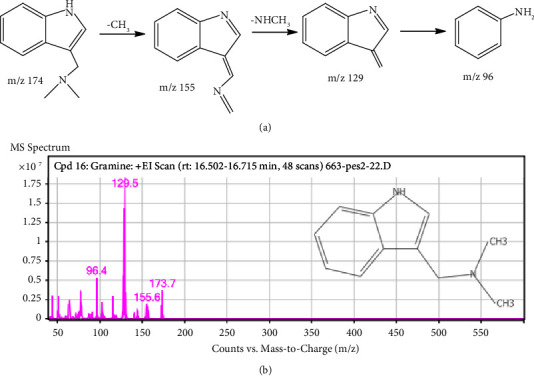
(a) Proposed fragmentation pathway and (b) mass spectrum of gramine.

**Figure 11:**
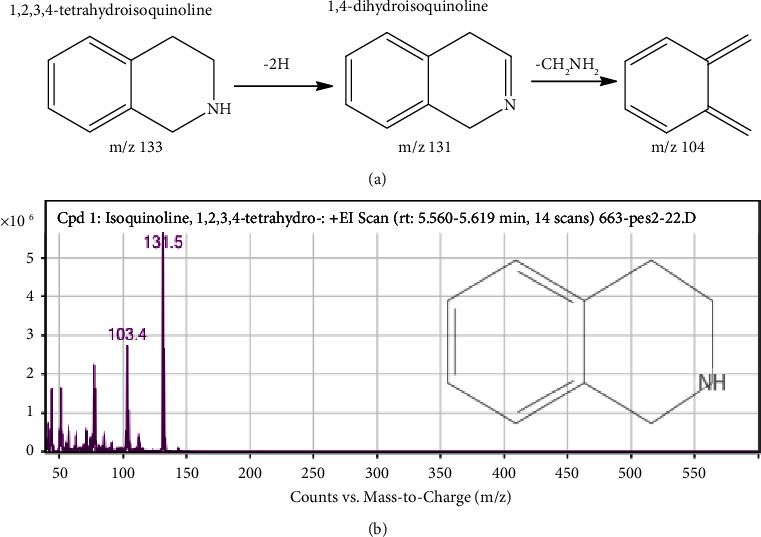
(a) Proposed fragmentation pathway and (b) mass spectrum of 1,2,3,4-tetrahydroisoquinoline.

**Figure 12:**
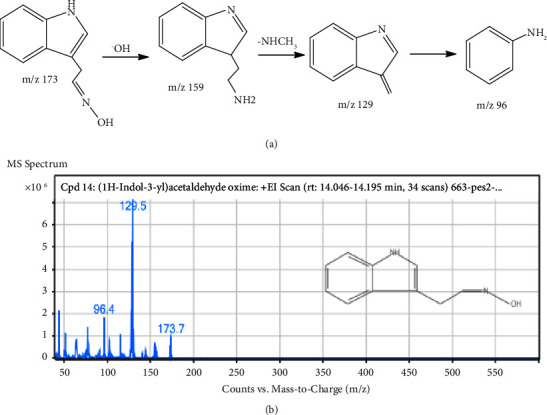
(a) Proposed fragmentation pathway and (b) mass spectrum of 1H-indol-3-yl acetaldehyde oxime.

The MS fragmentation pattern analysis of the alkaloidal extract (AAA) identified several alkaloidal derivatives (Figure 8). For instance, the most abundant peak (Figure 10, Table 2) was cautiously identified to be gramine, an aminoalkylindole alkaloid with a molecular weight and formulae of MW-174.24 gmol^−1^ [M^+^] and C_11_H_14_N_2_, respectively. This compound was fragmented by the loss of CH_3_· to produce a precursor ion at m/z 155.6. Further fragmentation gave a more stable molecule (Figure 10) to generate precursor ions at m/z 129.5 and 96.4 from the loss of amide (−26 Da), amine (−18 Da), and methyl groups (−15 Da) side chains after a possible 1,3 methyl McLafferty rearrangement of the dimethyl derivative of gramine to a more stable (E)-N-((3H-indol-3-ylidene)methyl)methanimine derivative (Figure 10(a)) [36]. Similarly, at retention times of 5.578 and 14.744 min, 1,2,3,4-tetrahydroisoquinoline and 1H-indol oxime derivatives were tentatively identified with molecular weights of m/z 131.5 and 173.7, respectively (Figures 11 and 12). The molecular formulae for these compounds are C_9_H_11_N and C_10_H_10_N_2_O, respectively (Table 2). The fragmentation of 1,2,3,4-tetrahydroisoquinoline led to the abstraction of two hydrogens to a more stable 1,4-dihydroisoquinoline (m/z 131.5), confirming the structure of the compound (Figure 11) [37]. In the case of 1H indole-oxime, the loss of –OH gave a precursor ion at m/z 159, followed by the loss of an amide and propyl groups to a molecular ion of m/z 129 and 96, respectively (Figure 12(a)).

**Figure 13:**
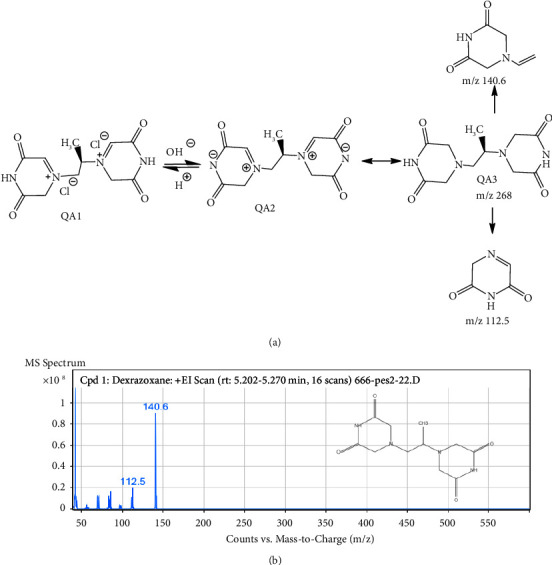
(a) Proposed fragmentation pathway and (b) mass spectrum of dexrazoxane.

The GC-MS profiles of phytocompounds from the quaternary alkaloidal extract (AAQ) of the *A. africana* plant also presented seven compounds. The major molecules were identified at RT 5.111 and 5.252 min (Figure 9) with a molecular ion at m/z 267.27 [M+]- (C_11_H_16_N_4_O_4_) and m/z 267.27 [M^+^]- (C_11_H_16_N_4_O_4_), respectively (Figure 13). Surprisingly, the molecular masses of these two compounds were the same, with a similar fragmentation pattern. These compounds were identified as dexrazoxane (85.59%) (C_11_H_16_N_4_O_4_), m/z 268.27, and its isomer razoxane (5.14%) (C_11_H_16_N_4_O_4_), m/z 268.27, a synthesized bisdioxopiperazine compound. It was determined that these two compounds were isomers of quaternary alkaloids in the form of QA1 and its zwitter-ionic form, QA2 (Figure 13(a)), similar to what has been reported by Rapoport and Holden [26]. The fragmentation pattern of this molecule at a higher CE level produced product ions at m/z 140.14 and 112.50, resulting from the cleavage of the tertiary carbon (Figure 13(a)). The rest of the compounds were in trace amounts (Table 3 and Figure 9).

## 4. Discussion

Plant medicine is largely prepared locally by decoction [38] or soaking in locally brewed alcohol for maximum infusion of phytoconstituents and *A. africana* is no exception. This plant has been an important folk remedy for people who have lived in mangrove-covered areas for a very long time. The paucity of data regarding the plant's antimalarial effects reinforces the need to investigate its antimalarial, antioxidant, and cytotoxic activities. Consequently, the extract yield in this study was within the predicted range of 1%–10% or more [39]. It is noteworthy that in an extraction process, the extract yield may be affected by several factors, including the type of solvent used, the extraction method employed, and the duration of the extraction [39]. In this study, the cold maceration extraction process was used for the extraction of the total crude (AAE). It was the most convenient and suitable method for the supposed thermolabile alkaloidal extracts inherent in the plant [39]. Several secondary metabolites have been identified in this plant in previous studies [17, 18], and such bioactive compounds have served as the foundation for the advancement and production of novel conventional drugs [8, 40].

In this study, crude alkaloids (AAA and AAQ) were extracted from the total crude extract (AAE) obtained from the leaves of *A. africana.* The extracts were evaluated for antiplasmodial activity using the SYBR Green I fluorescence assay. This method has been found not only to be fast, reliable, and relatively inexpensive but also to provide high-throughput screening of antimalarial drugs [4]. The SYBR Green I dye binds to the parasite's DNA in infected RBCs, resulting in high fluorescence that is detectable by flow cytometry.

The antiplasmodial properties of the alkaloidal extracts and the total crude extract yielded IC_50_ values that ranged from 6.217 to 11.890 *µ*g/mL in the order AAA < AAQ < AAE of activity. The results of this research suggest that the alkaloidal extracts (AAA = 6.217 *µ*g/mL and AAQ = 6.287 *µ*g/mL) showed better antiplasmodial effects than the total crude extract (AAE = 11.890 *µ*g/mL) as opposed to the reference drug (artesunate). Although the hydroethanolic extract of *A. africana* (AAE) showed good activity, as was similarly reported by Okokon et al. [41] for other plants, the alkaloidal extracts in our current study performed better and looked more promising. It further suggests that the antiplasmodial effects can be attributed mainly to the alkaloids present or may also be due to the synergy effect of the various compounds in this plant. Our findings are consistent with those of Erhunse et al. regarding the antimalarial effect of isoquinoline alkaloids such as berberine and palmatine [42]. Kyei et al. also demonstrated the potential antimalarial activity of cryptolepine and isocryptolepine [43]. The display of the potent antiplasmodial activity of the alkaloidal extracts in the present study confirmed earlier reports regarding the diversity of bioactive metabolites and, importantly, the antiplasmodial properties of several classes of alkaloids, such as the indole and isoquinoline classes [13, 44, 45].

The GC-MS profile of AAA revealed about nineteen phytocompounds (Table 2). A good number of compounds were predicted to be present in the extract used in this study. Notable among them are indole and isoquinoline alkaloids. Of the various compounds identified in the alkaloidal extract (AAA), gramine, a simple indole alkaloid, was found to have the highest percentage peak area of all the phytocompounds. Gramine has recently gained prominence due to its diverse biological activities, which include insecticidal, antibacterial, antiviral, antitumor, and anti-inflammatory properties [46]. It may be suggested that the antiplasmodial effects of the AAA extract in this study are largely due to the intrinsic indole alkaloids and isoquinoline alkaloids in the extract. Several indole alkaloids, including vinblastine and vincristine, have shown promising antimalarial effects. They are thought to disrupt the parasite's microtubule assembly, affecting its growth [47]. Tryptanthrin kills the parasite by interfering with the parasite's DNA synthesis [48]. Importantly, indole alkaloids are basic compounds that ionise in the acidic environments of the parasite's food vacuoles, contributing to their antimalarial activity. The quinoline ring was reported to have the potential to interfere with parasites' production of hemozoin, resulting in a buildup of toxic haem species that are harmful to the parasites. Also, some indole alkaloids have lipophilicity, which is a vital pharmacokinetic property that allows these drugs to penetrate the membrane of the parasite and interact with target sites [49].

Our current study supports previous research regarding the antimalarial properties of indole alkaloids isolated from natural products [50, 51]. It is important to note that indole alkaloids have long been suggested to possess a significant number of potent pharmacological properties, such as anti-inflammatory, cytotoxic, antiparasitic, antiviral, antagonistic, and serotonin-related activities [52]. Omar and colleagues reported on the antiplasmodial activities of two indole alkaloidal compounds, ellipticine and olivacine, that were isolated from plants of the genus *Aspidosperma* [49]. Another study found that isoquinoline alkaloids have antifungal, enzyme-inhibitory, antioxidant, antiviral, anticancer, antispasmodic [53], and antimalarial properties [54].

In addition, the GC-MS spectral analysis of the AAQ extracts revealed several compounds (Table 3). Among them were two enantiomorphic-pair compounds, dexrazoxane and razoxane (5.14%), with dexrazoxane having the highest (86%) peak area and the other five compounds having peak areas ranging from 0.93 to 3.09%. The antiplasmodial activity of the extract in this study may be due to the synergy of the various compounds or the single effect of dexrazoxane identified in AAQ. Dexrazoxane has been shown to have cardioprotective efficacy against doxorubicin-induced cardiotoxicity in breast cancer therapy [55]. Research has shown that the hydrolyzed product of dexrazoxane chelates with both bound and free iron [56], and the iron-chelator's strong iron binding properties may inhibit the intraerythrocytic development of plasmodium parasites [57].

Plasmodium metabolism is iron dependent. The iron-containing enzymes such as delta-aminolevulinate synthase and ribonucleotide reductase, which are needed for DNA synthesis, de novo haem production in the parasite, electron transport, and mitochondrial activity [58, 59], may have been deprived of iron by dexrazoxane in AAQ. It may be possible that the antiplasmodial effects of the AAQ extract are due to dexrazoxane. Dexrazoxane may have chelated with the irons required for the optimum function of these enzymes, which interferes with the parasite's metabolic activities and inhibits the intraerythrocytic growth process.

In natural product-based pharmacotherapy research for infectious diseases, the plant extract may possess high therapeutic efficacy but could cause potential harm to cells or organs; hence, screening for toxicity of promising or lead compounds is crucial. The current study evaluated the cytotoxic effects of the alkaloidal extracts and total crude extract using the MTT assay, as chronicled by Ayisi et al. [28]. This technique is based on the viable enzyme-mediated conversion of the yellowish tetrazolium to a purple compound (formazan) after reacting extracts with uninfected human erythrocytes. The CC_50_ values of AAA, AAQ, and AAE extracts were determined to be >100 *µ*g/mL in this study, and they have negligible cytotoxic effects on red blood cells (RBCs) [60]. A similar outcome was obtained for artesunate (the positive control drug). The high cell survival percentages recorded for alkaloidal extracts, total crude, and artesunate support the low toxicity or weak cytotoxicity to erythrocytes. The inability of plant extracts to cause the lysis of red blood cells *in vitro* may be highly connected to the inherent biological constituents in the plant, which ensure the protection of the erythrocytes against malaria parasite-mediated cellular damage [61]. The alkaloidal extracts (AAA and AAQ) and the total crude extract (AAE) gave good selectivity indices (greater than 2), which suggests that the extracts possess curative properties against *P. falciparum* parasites [62]. The determination of selectivity indices is key for evaluating the therapeutic potential of extracts in natural product drug discovery; it seeks to assess the relative safety as well as the efficacy of the plant extracts and their ability to target specific pathogens or cells, while at the same time, minimising any detrimental effects on normal cells.

The antioxidant properties of alkaloidal extracts and the total crude extract of *A. africana* were tested using various procedures based on assay principles and assay conditions [63]. The following assays were used in this study to assess antioxidant activity: total antioxidant capacity, DPPH radical scavenging activity, total glutathione, and ferric-reducing antioxidant power. The alkaloidal extracts and total crude extract demonstrated good antioxidant and reducing properties in a concentration-dependent manner. The display of scavenging abilities of the extracts for free radicals may be, to a greater extent, associated with the extracts' intrinsic properties [64]. The findings of this study on the antioxidant and antiplasmodial properties of isoquinoline and indole alkaloids, as well as several other compounds in the plant, agree with previous studies on alkaloids [65–68]. The artesunate reference drug used in this study had been reported to have antioxidant properties [69–72].

The reactive oxygen species (ROS) produced by oxidative stress-mediated damage to host RBCs and other organs during blood-stage schizogony may aggravate the infection. In addition, haemolysis produced by oxidative stress is a typical clinical occurrence after a few days of antimalarial therapy with artemisinin drugs and derivatives. The drug kills parasites by inducing ROS to be produced within infected red blood cells after the endoperoxide bridge is activated [73]. In this regard, it has been suggested that any potential antimalarial drugs should have scavenging properties to get rid of the extra free radicals generated by parasite metabolism and other exogenous factors [67, 74–76].

Generally, all the AAE, AAA, and AAQ extracts exhibited antioxidant properties in this study, and this may have contributed to the antiplasmodial activities recorded. Particularly, the dexrazoxane identified in AAQ, which is a known iron chelator, may be responsible for both the antiplasmodial and anticytotoxic effects on RBCs. Furthermore, the indole and isoquinoline alkaloids identified in the AAA extract potentially mediated the antiplasmodial and antioxidant activities in the current study. The pharmacodynamic profile of indole alkaloids and isoquinoline in malaria infection has been established [44]. The mechanism of dexrazoxane's antiplasmodial activity has been linked to iron but its anticardiotoxicity remains unknown.

The anticytotoxic and antioxidant properties of the extracts in this study may have contributed to protecting against the development of complications related to malarial infections. This may have also increased the efficacy of the extracts to promote rapid recovery. The identification of indole and isoquinoline alkaloids, as well as dexrazoxane compounds, in *A. africana* for the first time offers significant promise for advancing drug discovery and contributing to the development of novel therapies with potential advantages to human health. This study used the 3D7 *P. falciparum* strain for the antimalarial activity assay. Hence, other strains of the parasites are recommended to ascertain the effect of the alkaloids on them. The current study confirmed the antimalarial effects of these known indole and isoquinoline alkaloidal compounds. However, this is the first time these alkaloids, including dexrazoxane, have been identified in *A. africana* leaves.

We recommend *in vivo* studies of the alkaloidal extracts. Furthermore, we suggest that more studies be conducted by fractionating, isolating, and purifying these alkaloidal extracts to obtain pure phytocompounds for structure elucidation and optimisation studies. This will potentially introduce novel lead compounds from this plant into the antimalarial drug discovery pipeline.

## 5. Conclusion

The findings of this study suggest that the alkaloidal extracts of *A. africana* leaves possess promising antiplasmodial effects against 3D7 *P. falciparum* chloroquine-sensitive parasites. The results also showed that the *A. africana* leaves had antioxidant properties with negligible cytotoxic effects on erythrocytes. We acknowledge that the *in vitro* antimalarial study may not translate to clinical application as the protocol used has some limitations though the outcome of the study supports folklore application for malaria infection treatment. So far, this is the first time that isoquinoline alkaloids, indole alkaloids, as well as razoxane and dexrazoxane have been identified in *A. africana* leaves to the best of our knowledge.

## Figures and Tables

**Table 1:** Antiplasmodial, cytotoxic activities, and therapeutic indices of the AAE, AAA, and AAQ extracts of *A. africana*.

Extracts	Antiplasmodial efficacy against 3D7 *P*. *falciparum* IC_50_ ± SD (*µ*g/mL)	Cytotoxicity against RBCs CC_50_ (*µ*g/mL)	Selectivity indices CC_50_/IC_50_
Crude extract (AAE)	11.890 ± 0.011^*∗∗*^	>100	>8.410^*∗∗*^
Crude alkaloids (AAA)	6.217 ± 0.012^*∗∗*^	>100	>16.085^*∗∗*^
Quaternary alkaloids (AAQ)	6.285 ± 0.456^*∗∗*^	>100	>15.910^*∗∗*^
Artesunate (control)^*∗*^	0.09 ± 0.03 (×10^−3^)	>100	>10000

^*∗*^Artesunate was utilized as the reference drug. The data show averages for duplicate runs ± SD (standard deviation). Differences in mean values that are statistically significant (*p* < 0.01) were shown using the symbols (^*∗∗*^).

**Table 2:** GC-MS profiles of phytocompounds of the crude alkaloidal extract (AAA) of 70% ethanol leaf extract of the *A. africana* plant.

S. nos.	RT (min)	Name of compound	MF	MW (g/mol)	% Peak area
1	5.578	1,2,3,4-Tetrahydroisoquinoline	C_9_H_11_N	133.19	2.28
2	5.850	N-[3-[N-Aziridyl] propylidene] tetrahydrofurfurylamine	C_10_H_18_N_2_O	182.26	0.61
3	6.058	(+)-Dibenzoyl-L-tartaric acid anhydride	C_18_H_12_O_7_	340.30	2.47
4	6.140	3-Phenyl-3-pentanol	C_11_H_16_O	164.24	1.05
5	6.643	7-Methyl-2-oxa-7-azatricyclo [4.4.0.0(3,8)] decane	C_9_H_15_NO	153.22	2.47
6	6.810	Hydrazine, 1-(2-ethyl-6-methylphenyl)-	C_9_H_14_N_2_	150.22	2.92
7	7.626	2-Phenyl-l-p-toluene esulfonylaziri-dine	C_15_H_15_NO_2_S	273.40	1.15
8	7.952	Benzenepropanoic acid, octyl ester	C_17_H_26_O_2_	262.40	2.57
9	8.020	2,5-Octadecadiynoic acid, methyl ester	C_19_H_30_O_2_	290.40	1.75
10	9.999	2-Propenoic acid, 3-phenyl-	C_9_H_8_O_2_	148.16	4.95
11	10.163	Ethanone, 1-hydroxy-2,6,6-trimethyl-2,4-cyclohexadien-1-yl-	C_11_H_16_O_2_	180.24	3.19
12	12.808	5,5,8a-Trimethyl-3,5,7,8,8a-hexahydro-2H-chromene	C_12_H_20_O	180.29	4.83
13	13.221	2H-Oxecin-2-one, 3,4,7,8,10-hexahydro-4-hydroxyl-10-methyl-,[4s-(4R∗,5E,10S∗)]-	C_10_H_16_O_3_	184.23	13.11
14	14.127	(1H-indol-3-yl) acetaldehyde oxime	C_10_H_10_N_2_O	174.20	8.10
15	14.744	(1H-indol-3-yl) acetaldehyde oxime	C_10_H_10_N_2_O	174.20	7.04
16	16.683	Gramine	C_11_H_14_N_2_	174.24	31.97
17	22.139	7-Nonenamide	C_9_H_17_NO	155.24	3.43
18	26.139	8,11,14-Eicosatrienoic acid, (Z, Z, Z)-	C_20_H_34_O_2_	306.50	2.39
19	28.721	4-Nitro-benzoic acid,1-methyl-heptyl ester	C_15_H_21_O_4_	279.33	3.38

GC-MS = gas chromatography-mass spectrometry, RT = retention time (mins), MF = molecular formula, MW = molecular weight, and AAA = crude alkaloids of *A. africana *leaf.

**Table 3:** GC-MS profiles of phytocompounds of the quaternary alkaloidal extract (AAQ) of 70% ethanol leaf extract of the *A. africana* plant.

S. nos.	RT	Name of compound	MF	MW	% Peak area
1	5.111	Razoxane	C_11_H_16_N_4_O_4_	268.27	5.14
2	5.252	Dexrazoxane	C_11_H_16_N_4_O_4_	268.27	85.59
3	7.001	2-Amino-3-(hydroxyphenyl)-propanoic acid	C_9_H_11_NO_3_	181.19	1.90
4	7.059	1-Gala-1-ido-octose	C_8_H_16_O_8_	240.21	1.15
5	7.861	Benzoic acid, 3-(diethylamino)-, methyl ester	C_12_H_17_NO_2_	207.27	1.04
6	13.171	2H-Oxecin-2-one, 3,4,7,8,9,10-hexahydro-4-hydroxy-10-methyl-, [4S-94R*∗*,5E,10S*∗*]-	C_10_H_16_O_3_	184.23	3.09
7	16.556	(1H-indo-3-yl) acetaldehyde oxime	C_10_H_10_N_2_O	174.20	0.93

GC = gas chromatograph, RT = retention time (min), MF = molecular formula, MW = molecular weight, and AAQ = quaternary alkaloids of *A. africana *leaf.

## Data Availability

The data used to support the findings of this study are included within the article.

## Abbreviations

ART: Artesunate
ACT: Artemisinin-based combination treatment
AAA: Crude alkaloids
CPM: Complete culture medium
CC_50_: Cytotoxic concentration
DCM: Dichloromethane
DPPH: 2,2-diphenyl-1-picrylhydrazyl assay
EDTA: Ethylenediaminetetraacetic acid
EC_50_: Effective concentration at 50%
Fe^2+^-TPTZ: Ferrous tripyridyltriazine
Fe^3+^-TPTZ: Ferric tripyridyltriazine
FRAP: Ferric-reducing antioxidant power
GC-MS: Gas chromatography-mass spectrometry
GC: Gas chromatograph
GSH: Glutathione
IC_50_: Inhibition concentration
MF: Molecular formula
MW: Molecular weight
NIST: National Institute of Standards and Technology
AAQ: Quaternary alkaloids
RBCs: Red blood cells
RT: Retention time
ROS: Reactive oxygen species
SD: Standard deviation
TAC: Total antioxidant capacity
GSH: Total glutathione concentration
AAE: Total crude extract
WHO: The World Health Organization
MTT: The tetrazolium-based colorimetric
TLC: Thin layer chromatography.
